# Evaluation of Glycemic Index of Six Different Samples of Commercial and Experimental Pasta Differing in Wheat Varieties and Production Processes

**DOI:** 10.3390/foods10092221

**Published:** 2021-09-18

**Authors:** Amalia Pandolfo, Bernardo Messina, Giuseppe Russo

**Affiliations:** Gian Pietro Ballatore Research Consortium, Z.I. Dittaino I, 94010 Assoro, EN, Italy; pandolfo.amalia@gmail.com (A.P.); info@ilgranoduro.it (B.M.)

**Keywords:** pasta, glycemic index, glycemic response, durum wheat, landraces

## Abstract

Pasta is a staple food of the Mediterranean Diet, and it is traditionally made of durum wheat semolina. In Sicily, durum wheat production and its transformation into semolina, bread, and pasta are well-developed economic sectors. For pasta, there is a wide supply of commercial brands, whether coming from conventional industrial manufacturing or from medium to small and local handcrafted production. Both conventional durum wheat and local durum wheat landraces, such as *Timilia* and *Russello*, are used for pasta production, but local landraces are, for the most, transformed into handcrafted pasta. The market of local landraces durum wheat pasta has risen in recent decades, in Sicily and in Italy as well, boosted by a perceived high nutritional and healthy value of these wheat derivatives. In particular, a popular and scientifically unproven idea suggests that a reduced glycemic response might be elicited by these pasta landraces. Therefore, to test this hypothesis, the main objective of the present study was the evaluation of the glycemic index (GI) of four samples of *Timilia* and *Russello* handcrafted pasta and two samples of conventional durum wheat pasta. The study enrolled fourteen healthy weight male and female volunteers aged from 18 to 46; eight test sessions were performed twice a week, every session testing a pasta sample (six sessions) or the glucose solution chosen as reference food (two sessions). The standard methodology for GI measurement was followed during each step of the study. The six tested pasta samples were characterized regarding their composition (protein, fiber, and starch content) and their whole production processes (milling method and milling diagram of flour or semolina, drying temperature, and diagram of pasta shape). The six tested pasta samples showed GI values ranging from low (34.1) to intermediate (63.1). *Timilia* and *Russello* pasta are the first GI calculations available. The two samples made of conventional grains showed lower values of GI (34.1 and 37.8). The results do not support the popular idea of a reduced glycemic response elicited by *Timilia* and *Russello* wheat landrace pasta; the tested samples showed GI values in the range of 56.2 to 63.1. However, some consideration should be made of factors other than wheat varieties and related to production processes that may have affected the final GIs of the pastas. Even if the study is not designed to discriminate among factors related to wheat varieties or processes used to produce different pasta, it is a preliminary step in the characterization of the healthy potential of the local wheat landraces, popularly called ancient grain. A future implementation of the local wheat landraces supply chain should pay attention to all the factors above, from a better seed identity certification to the production process in order to further improve the healthy value of these staples of the Mediterranean Diet.

## 1. Introduction

The glycemic index (GI) was introduced in 1981 [1] as a way of classifying carbohydrate-rich foods according to their effects on postprandial glycemia.

In detail, the GI measures the postprandial blood glucose response of a 50 g portion of available carbohydrate as a percentage of the blood glucose response elicited by 50 g of a reference carbohydrate, such as glucose or white bread [1,2].

For both test and reference foods, the Incremental Area Under the Curve (IAUC) of blood glucose concentration in two hours is calculated. The ratio percentage of the two areas is the GI for the tested food [2,3,4].

For a starchy food, there are many factors able to more or less make the enzymatic release of its glucose content in the intestinal lumen and/or its absorption easy, leading to a major or minor increase in blood glycemia of a subject, regardless of their physiological background.

For pasta, the focus food in this study, durum wheat varieties, pasta composition (fiber, protein, and starch content), structure and dimensions of the raw starch granules, milling processes and flour or semolina grinding diagram, drying temperature and diagram, pasta shape, starch gelatinization degree after cooking and cooking time [5,6,7,8] are all factors affecting the glycemic response after pasta ingestion, and as a consequence, its final GI [9,10].

Since its introduction, several papers have been published pointing out a method for determining GI of different foods [2,3,4] and assessing the health impact of diets based on low, moderate, or high GI foods on the outcome, such as metabolic syndrome, diabetes prevention and control, prevention of cardiovascular disease (CVD), reduction and prevention of obesity, and prevention of some cancers, i.e., breast and colon cancers [5,9,10,11]. One point for wide and useful use of GI and GL in dietary recommendations is the availability of reliable GI data for the food items actually consumed by people in a specific country.

Most of the existing tables report the calculated GI for foods usually available and eaten in the United States or other Western countries [1,12,13], whilst less GI data has been collected and reported for foods usually manufactured or eaten in the Mediterranean countries and in Italy.

A recent contribution that has partially filled the gap is the paper of Scazzina et al. (2016), reporting the GI of 124 different food items easily available in Italian markets [14].

The current study evaluated for the first time the GI of four pasta samples, produced from durum wheat landraces cultivated in Sicily (*Russello* and *Timilia*), using the standard methodology for GI measurement.

In addition, two other samples obtained from industrial pasta factories, one commercial wholemeal pasta and one experimentally produced pasta (not wholemeal), were added to the study.

*Timilia* and *Russello* Sicilian wheat landraces, locally named “ancient grain,” were chosen because of the recent sharp increase in their local customer demand, related to a hypothetical higher healthy value of their derivatives [15,16,17,18,19,20,21].

Namely, it is generally assumed that derivatives of these traditional landraces might elicit a reduced glycemic response, but, thus far, there is no scientific data supporting this specific view.

The study also collected data on tested pasta composition and on the processes for their production, as well as an attempt to draw attention to variables that may have affected the final GI values measured in the study.

Therefore, the study should be considered a contribution to a better characterization of local wheat landrace derivatives.

Moreover, it points to classify the six pasta samples analyzed according to Ramdath [5], which defines food containing starch or carbohydrates as “High” (GI range from 100 to 70), “Intermediate” (GI range from 69 to 65), and “Low” (GI less than 55) glycemic index classes.

## 2. Materials and Methods

### 2.1. Study Design

The glycemic indexes (GIs) of 4 Timilia and Russello handcrafted pasta samples and 2 samples of conventional durum wheat pasta were evaluated. The GI was determined according to Wolever (2010), Brouns et al. (2005), and the FAO/WHO (1998) method and procedures [2,3,4]. A total of 14 healthy weight male and female volunteers, aged from 18 to 46 were enrolled in the study, and 8 test sessions were performed twice a week, every session testing a pasta sample (6 sessions) or the glucose solution chosen as reference food (2 sessions).

### 2.2. Logistic and Protocol Requirements

The hotel management school, *Istituto Professionale di Stato per i Servizi Alberghieri e della Ristorazione “Paolo Borsellino”* (Palermo, Italy), made available suitably equipped rooms and a professional kitchen for the study; the kitchen staff was trained to cook and serve each sample according to the study design.

A team of 2 researchers supervised the tests and collected field data. In total, 8 evaluation tests were scheduled, 1 for each pasta sample and 2 additional sessions for reference food (50 g of glucose in 250 mL solution). On each test day, a single sample, pasta or reference food, was tested on every volunteer.

The volunteers fasted overnight and were instructed to keep their usual dietetic habit and to repeat the same standardized dinner by 7:00–8:00 p.m., each evening before the tests. No alcohol or intense physical activity was allowed the day before the test. The test started at 7:00 a.m. and ended at 9.30 a.m.

During the 6 pasta testing sessions, subjects were asked to drink 250 mL of mineral water provided with the serving (labeled “Acqua Vera”). They were asked to consume the sample and drink the water in not more than 10 min, and to stay sitting for the next 2 h without eating or drinking anything.

Three specialized nurses took care of sampling fingertip capillary blood at 0 (fasted state) and at 15, 30, 45, 60, 90, 120 min from food ingestion. Glucometers (ACCU-CHEK-AVIVA; labeled Roche Diagnostics GmbH, Mannheim, Germany) were used to measure blood glucose concentration (mmol). The choice of the glucometer device was settled by suggestions of Freackman et al. 2012 [22] that argued the best type of systems available for guaranteeing accuracy evaluation according to DIN EN ISO 15.197: 2003.

The measured glycemia values were used to build the curve of glycemic response for every volunteer and for each tested and reference food.

### 2.3. Healthy Volunteers

Totally, 29 subjects from the general population responded to a public claim for trial recruiting. Before recruiting, each one of the subjects had an interview with a physician and an accredited dietitian to verify their good health and suitability to the purpose of the study.

For each of them, weight, height, and body mass index (BMI) were measured and calculated. Volunteers were individually instructed on the aim of the study, on the protocol that would be applied, and informed about the risks of the procedure. Of the 17 initially recruited subjects, only one was labeled as IG15 withdrawn, while the other two subjects (IG09 and IG14) were discarded from the study, having missed some of the test sessions. Therefore, final data were available for 14 subjects who took part in the 8 scheduled test sessions from 10 November 2015 to 4 December 2015.

The sessions were performed twice a week, allowing at least 48 h of wash-out between 2 following test days. Only 14 volunteers completed every session of the study. The volunteers were adult males (*n* = 14) and females (*n* = 3) aged from 18 to 48 years. A label from IG01 to IG17 was assigned (Table 1). As reported and explained in the results, just 3 of the volunteers withdrew from the study.

### 2.4. Tested Food

The trial was designed to measure the GIs of 6 different types of pasta, whose characteristics are reported in detail in Table 2. The 6 samples were labeled as follows:

- ICS experimental pasta (shape spaghetti) produced by the industrial plant using a mix of varieties of durum wheat cultivated in Sicily (*Duilio*, *Simeto*, *Iride*, and *Saragolla*).
- COMM commercial wholewheat pasta (shape spaghetti) produced by means in an industrial plant using an unknown mix of varieties of durum wheat.
- TI-DIMESA experimental handcrafted pasta (shape tagliatelle) from *Timilia* Sicilian landrace.
- TI-PONTE commercial handcrafted pasta (shape tagliatelle) from *Timilia* Sicilian landrace.
- RU-DIMESA experimental handcrafted pasta (shape tagliatelle) from *Russello* Sicilian landrace.
- RU-PONTE commercial handcrafted pasta (shape tagliatelle) from *Russello* Sicilian landrace.

As reference food, a solution containing 50 g of glucose in mineral water for 250 milliliters of volume (water labeled as “Acqua Vera”) was chosen. The reference food was prepared and bottled by accredited chemists (Farmacia Sanfilippo-Palermo).

The reference food was tested twice, while each sample of pasta was tested once for each volunteer. For each type of pasta previously calculated, the sample serving size contained 50 g of available carbohydrates, and cooking time was tested (Table 3). Indeed, cooking time is well known as an important parameter affecting the pasta GI, and is often reported in official GI tables of food.

The kitchen staff and one of the researchers had previously created the needed tests to calculate the optimal cooking time for each pasta sample.

Unsalted boiling water was chosen to cook the pasta to the preferred cooking point in Italy named “cottura al dente,” corresponding to the time when samples cooked were firm to the bite and not sticky during chewing [23].

At this point, the portions of pasta were quickly served without any sauce.

Pasta ICS is an experimental product shaped as spaghetti and made by a mix of semolina from four “modern” varieties of Sicilian durum wheat, namely *Duilio*, *Iride*, *Saragolla*, and *Simeto*.

Pasta ICS production took place at the factory *Molino e Pastificio Tomasello*, in Trabia (Palermo, Italy). The semolina used to produce pasta ICS showed a specific grinding diagram characterized by the total removal by extraction of the so-called “farinette” fraction, made up of particles with a diameter ≤118 µ, namely the less sized fraction obtained after milling durum wheat (according to UNI 10873:2000, which is the Italian standard to determine and classify durum wheat Semolina granulometry) [24]. As the last step of its production process, pasta ICS underwent a dynamic drying process, which used a temperature diagram always over 80–90 °C.

Pasta COMM is a wholegrain commercial pasta (spaghetti) available in a well-known Italian large-scale retail distribution. The shape was spaghetti, and the sample was produced with the pasta factory industrial method, not handcrafted. No other information about durum wheat varieties or milling diagrams was available for this sample.

TI-DIMESA e TI-PONTE are 2 wholegrain samples of pasta, shaped as tagliatelle and obtained from an artisanal production process. The flour was obtained by stone milling durum wheat of landrace *Timilia*. The flour was analyzed in the laboratory to evaluate the particle size profile using a Buhler automatic sieve. The analysis results showed that more than 20% of the flour particles had a diameter ≤118 µ. The 2 *Timilia* pasta samples underwent a drying process with a low-temperature drying diagram (30 °C or 40 °C) with a static drying cell.

In addition, RU-DIMESA e RU-PONTE are 2 wholegrain samples of pasta, shaped as tagliatelle, made up from a stone-milled flour of durum wheat landrace, *Russello*. This flour was analyzed in the laboratory to evaluate the particle size profile by Buhler automatic sieve confirming the presence of particles with a diameter ≤118 µ at more than 20% of the total. The *Russello* tagliatelle pasta was obtained from an artisanal production process and dried by a low-temperature drying diagram (30 °C or 40 °C) with a static drying cell.

Official methods were used to analyze every pasta sample to determine protein content (Kjeldhal AACC 47-12), moisture (UNI EN ISO 712:2010), total dietary fiber (Megazyme kit based on AACC method 32-05.01 and AOAC method 985.29), and resistant and total starch content (Megazyme kit K-TSTAR based on AACC 76.13).

### 2.5. Data Collection and Statistical Analysis

The measured glycemic values were used to build the curve of glycemic response for every volunteer and for every tested food, including the reference one. Then, for each sample and each study subject, the incremental area under the blood glucose response curve (IAUC) was calculated geometrically, using the trapezoid rule, and ignoring the area below the fasting baseline. The GI calculation for each pasta sample used the method referred to as the mean of the ratios. For each subject, the ratio between the individual IAUC after consuming the pasta sample and the IAUC for the same subject after consuming the reference food was calculated, expressed as a percentage value. Then, the GI of each pasta type was calculated as the average value of the ratios across all the subjects consuming the pasta sample [2,3,4]. One-way analyses of variance (ANOVA) were used to compare differences between the eight evaluation sessions with post-hoc Tukey HSD test. A *p*-value ≤0.05 was considered statistically significant. Pairwise comparison of glycemic index among all pasta samples ANOVA was also carried out.

## 3. Results

The results revealed, for the six tested samples, GI values ranging from 34.1 to 63.1 (Table 4). The lowest GI (34.1) was obtained for the pasta ICS, followed by pasta COMM, which showed a GI value of 37.8. According to the current classification [5], both these pastas should be considered low GI starchy foods. Pasta COMM is a wholegrain sample likely obtained from an industrial process as the ICS sample, but we have no details about durum wheat varieties used for its production. The other four pastas made with landraces Timilia (TI-DIMESA and TI-PONTE) or Russello (RU-DIMESA and RU-PONTE) showed higher GI values ranging from 56.2 to 63.1, making these pasta assessed as intermediate GI foods.

Three of the samples, in the order of RU-PONTE, TI-DIMESA and RU-DIMESA, showed close values (56.2, 57.1, and 59.2) and just the sample TI-PONTE (Timilia) registered the higher value of 63.1, which was also the highest calculated in the study and the higher value if compared with recent data recorded for Italian pasta [14].

Post-hoc analysis for pairwise comparison (Table 5) showed statistically significant higher GI values of TI-PONTE compared to ICS (difference = 29.0; 95% C.I. 6.1~51.9; *p*-value = 0.005) and COMM (difference = 25.3; 95% C.I. 2.0~48.6; *p*-value = 0.026), higher GI values of RU-DIMESA compared to ICS (difference = 25.1; 95% C.I. 2.2~48.0; *p*-value = 0.024) and TI-DIMESA compared to ICS (difference = 23.0; 95% C.I. 0.1~45.9; *p*-value = 0.049). Differences in GI values resulted in marginally significant differences between RU-DIMESA and COMM (difference 21.4; 95% C.I. −2~4.7; *p*-value = 0.092) and between RU-PONTE and ICS (difference 22.1; 95% C.I. −0.8~45.0; *p*-value = 0.092).

For each pasta sample, the ICS, COMM, RU-DIMESA RU-PONTE, TI-DIMESA, and TI-PONTE, and for each reference food session, data of the average glycemic response (mmol /L) after food ingestion, recorded at zero (t0), 15, 30, 45, 60, 90, and 120 min, were reported (Table 6 and Table 7).

The analysis of variance of the glycemic indexes showed a significant difference in the mean values for the different types of pasta (*p*-value < 0.001; Table 8). 

The analysis of variance (ANOVA) showed that, excluding time at 0 (T0) and 90 min (T90), there was always a significant difference between the mean values of glycemia in each sampling time (six pasta samples and two reference foods). 

Post-hoc pairwise comparison analysis showed statistically significant higher glycemia values of the RU-PONTE versus COMM (difference +1.04 mmol; 95% C.I. 0.25~1.82; *p*-value = 0.003) and versus ICS (difference +0.99 mmol; 95% C.I. 0.23~1.76; *p*-value = 0.003) at 30 min (T30); difference between RU-PONTE and TI-PONTE was marginally significant (difference +0.74 mmol; 95% C.I. −0.03~1.50; *p*-value = 0.067). RU-PONTE glycemic values resulted in being statistically higher with respect to COMM (difference +1.06 mmol; 95% C.I. 0.09~2.03; *p*-value = 0.023) and ICS (difference +0.96 mmol; 95% C.I. 0.01~1.91; *p*-value = 0.047) at 45 min (T45). 

The two reference food curves did not show any significant differences at every time point.

## 4. Discussion

The results obtained from the study revealed a lower GI for industrial pasta ICS and COMM (34.1 and 37.8) and higher GIs for handcrafted landraces (*Timilia* and *Russello*) pasta (RU-PONTE, RU-DIMESA, TI-PONTE e TI_DIMESA). By applying the standard GI measurement methodology, this study produced unique data for pasta made from durum wheat landraces cultivated in Sicily. The results do not support the popular idea of a reduced glycemic response elicited by *Timilia* and *Russello* wheat landrace pasta. Further investigations are needed to clarify the supposed healthier value of “ancient grain” based food.

The meager data available for GI of Italian pasta [14] revealed that values higher than 55 were not common for durum wheat pasta made without other ingredients and additives. On the other hand, low values (below 35) were mainly expected for wholemeal pasta, with some exceptions. Our results were partially in line with these data.

The four samples obtained from Sicilian landraces (RU-PONTE, RU-DIMESA, TI-PONTE e TI_DIMESA) showed values ranging from 56.2 to 63.1. Although these samples were made with wholemeal flour, usually associated with lower GI [25,26,27,28], a higher GI was recorded. It is known that the presence of fiber may result in an unstructured pasta matrix, leading to a more rapid digestion of starch and, subsequently, to higher GI [29,30]. These data confirm that it is necessary to clarify the role of the fiber content in reducing GI, in moderating the rate of glucose absorption and controlling the glucose response curve.

It is hypothesized that other process variables make the GI higher with a more relevant impact with respect to the fiber content. Among these variables could be considered the fine grain size, which characterizes the stone-milled flour [5,6,7,8], the low-temperature used for drying processes [31], the low gluten index that characterizes Sicilian landraces [19], and the probable presence of soft grains in the samples of Sicilian landraces [32]. All these variables contribute to making GI higher.

The stone-milled flours used to produce experimental or commercial samples of pasta from *Timilia* and *Russello* landraces were characterized by a large fraction of “*farinette*” (more than 20% of particles of flour with a diameter ≤118 µ), and it could be related to a higher GI and may have a relevant impact on starch availability to be depolymerized [5,6,7,8]. Indeed, the traditional stone milling of grains can consistently damage the cell wall structure, increasing the exposure of the entrapped starch granules to enzymatic digestion [7,8,33].

The stone milling process is prone to damage the cell walls, which may have in turn affected the gelatinization degree of starch granules during cooking [26,34,35,36]. The degree of starch damage resulting from milling is one of the factors known to increase the starch gelatinization degree during wet cooking [37,38,39,40,41].

The results about pasta ICS can be discussed by considering the variables that produced this type of experimental sample, which showed the lowest GI (34.1) compared to the other samples of the study, which were all wholemeal.

Pasta ICS was an experimental pasta made of a mix of modern semolina wheat varieties whose peculiar characteristic was the total removal of the “*farinette*” fraction. The steel roll milling of the wheat varieties used for pasta ICS, instead, gave mixed semolina characterized by larger and (probably) less damaged particles, a trait magnified by the total experimental removal of the *farinette* fraction.

The comparable sample named COMM, a wholemeal commercial Italian pasta (spaghetti) showed a slightly higher GI value despite its fiber content (37.8).

For the pasta COMM sample, a low GI value would be expected due to its high fiber content.

Fiber content, an otherwise healthy and recommended element of the diet, in this study seems to not be strongly related to the GI values of the samples. It should also be considered that the total dietary fiber content for the samples was estimated without discriminating between the insoluble/soluble fractions, which might have had a different impact on the GI of food [25,26,27,28].

There could be a relationship, instead, between the grinding diagrams of flours or semolina used to make pasta and the GI results of the samples. In further studies, the role of stone milling processes versus steel roll milling could be compared in order to evaluate the impact on GI of these different grinding methodologies.

There are at least two other factors we may consider relevant to determining a low GI for pasta ICS, compared to the intermediate GI for *Timilia* and *Russello* pasta samples, namely the drying temperature diagram and the wheat varieties used.

For traditional Sicilian landraces, *Timilia,* and *Russello* pasta samples, the protein content recorded was around 10% (see Table 2), whilst it was over 12% for pasta ICS and pasta COMM. Sicilian landraces are known for being characterized by a lower gluten index (a widely accepted indicator of gluten strength) if compared to “modern” varieties [19]. The different protein content and mostly gluten strength are important factors for determining the tighter or less tight interaction between the starchy matrix and the gluten net in the dough and in the final product [29,39].

This aspect may have been amplified by the low-temperature (less than 40 °C) drying process submitted by *Timilia* and *Russello* pasta samples, resulting in a different texture of pasta and in a leakier interaction of starch with the gluten net.

On the other hand, drying pasta at high temperature (90 °C), as it was conducted for pasta ICS and probably pasta COMM, contributed to protein aggregation and to further strengthen the protein network around the starch, reducing its digestibility [29].

The gluten weakness for the landrace’s pasta samples could be explained by the releasing of a greater amount of starch in the cooking water compared to the ICS and COMM samples (see Table 3), supporting the hypothesis for Sicilian landraces wheat pastas of a starch matrix less effectively trapped in the gluten net. Indeed, the number of servings for different samples needed to be adjusted after the preliminary cooking tests.

Another reason to explain the higher GI values recorded for Sicilian landrace pasta samples may be found in the accidental and common contamination of *Timilia* and *Russello* crops with a certain amount of soft wheat due to its presence in seed grains and in saw bed [23]. The higher ratio amylopectin/amylose, typical of soft grain-derived starches, could partially explain the intermediate GI values recorded for landrace pasta [12,13].

In summary, according to the collected data and the study results, it was hypothesized that each one of the different pasta production steps, i.e., the milling procedures of the grain, the drying temperature diagram and the choice of a specific wheat variety, should be considered relevant in affecting the GI values of the final products.

## 5. Conclusions

The commonly held belief that ancient grains hold a higher health value than conventional or modern variety grain chains is not supported in this GI study involving pasta samples, regardless whether these products are handcrafted, stone-milled wholemeal flours. Pasta samples obtained from landraces always showed a higher GI value if compared to other experimental or commercial samples.

However, it is plausible to hypothesize that by acting in a controlled manner on specific process variables, the result of the production of a low GI type of pasta can be pursued. It is possible to act on production processes, to control variables as wheat varietal selection, cultivation techniques, gluten strength with the protein content, grinding diagram and typology of the milling process, extrusion pressure [42] of the pasta production and shaping, in order to effectively reduce the GI of pasta, and maybe other wheat derivatives, without altering their original formula by the addition of extra ingredients.

In this contest, however, it is necessary to clarify which process variables have major impacts on determining a lower glycemic response and a low GI in order to transfer to the pasta factories the technological protocols for the production of innovative low-GI pasta. The fact that the presence of dietary fiber does not always correspond to a lowering of the GI value has also been confirmed.

Greater efforts must be made to understand how the choice of a grinding diagram that eliminates “farinette” fraction from semolina, or the use of high temperatures in the drying processes, can lead to a lowering of the GI.

## Figures and Tables

**Table 1:** Data on volunteers enrolled in the study (BMI = Body Mass Index).

Label	Gender	Age	Weight (kg)	Height (cm)	BMI
IG01	M	18	69.5	166	25.0
IG02	M	19	78	178	25.0
IG03	M	32	73	175	23.8
IG04	F	26	64	170	22.1
IG05	F	46	67	167	24.0
IG06	M	26	98	195	25.8
IG07	M	26	80	177	25.5
IG08	M	19	91.5	188	25.5
IG10	M	25	74	170	25.6
IG11	M	25	72	175	23.5
IG12	M	45	71	174	23.5
IG13	F	43	54	158	21.6
IG16	M	40	85	181	25.9
IG17	M	38	94	193	26.0
Mean		30.6	76.2	176.2	24.5
SD		10.0	12.7	10.4	1.4

**Table 2:** Characteristics of samples of pasta analyzed.

Pasta Sample	Varieties or Landraces	Whole Wheat Pasta	Milling Process	Note on Flour	Drying Process	Protein Content (%)	TDF (%DM)	RS (%DM)
ICS	Mix of modern varieties *Duilio*, *Simeto*, *Saragolla*, *Iride*	NO	Cylinder grinding	Semolina totally deprived of “farinette” fraction ^1^	Dynamic, at high temperature (90°)	12.50	4.65	0.76
COMM	Mix of unknown modern varieties (commercial pasta)	YES	Unknown	Whole Semolina	Unknown	12.00	6.50	0.47
RU-DIMESA	*Russello* Sicilian landrace	YES	Stone milling	More than 20% of “farinette” ^1^	Static, at temperature around 30 °C	11.24	8.43	0.68
RU-PONTE	*Russello* Sicilian landrace	YES	Stone milling	More than 20%of “farinette” ^1^	Static, at temperature under 40 °C	10.50	9.68	0.23
TI-DIMESA	*Timilia* Sicilian landrace	YES	Stone milling	More than 20% of “farinette” ^1^	Static, at temperature around 30 °C	10.83	8.13	0.48
TI-PONTE	*Timilia* Sicilian landrace	YES	Stone milling	More than 20% of “farinette” ^1^	Static, at temperature under 40 °C	10.60	9.61	0.51

^1^ We consider as “farinette” the fraction of flour with a particle diameter ≤118 µ. TDF = Total Diet Fiber, RS = Resistant Starch, DM = Dry Matter.

**Table 3:** Scheme to evaluate 50 g of available carbohydrates for each sample serving.

Pasta Samples		Cooking Time Minutes		Total Starch % Dry Matter	Total Starch %	Serving Containing 50 g Available CHO
ICS	raw		raw	76.39	70.81	70.60
	cooked	13	cooked	78.23	71.43	70.00
COMM	raw		raw	72.63	64.03	78.08
	cooked	11	cooked	73.69	67.32	74.27
RU-DIMESA	raw		raw	70.28	63.52	78.71
	cooked	8	cooked	70.08	64.43	77.61
RU-PONTE	raw		raw	69.89	61.15	81.75
	cooked	9	cooked	66.57	39.15	127.71
TI-DIMESA	raw		raw	67.43	60.21	83.03
	cooked	8	cooked	57.77	52.40	95.43
TI-PONTE	raw		raw	74.58	65.22	76.66
	cooked	9	cooked	70.37	40.90	122.25

**Table 4:** Glycemic Index evaluated for the six pasta samples.

Samples	GI	SD
ICS	34.1	15.2
COMM	37.8	21.8
RUDIMESA	59.2	21.2
RUPONTE	56.2	18.0
TIDIMESA	57.1	27.4
TIPONTE	63.1	18.6

SD = Standard Deviation.

**Table 5:** Pairwise comparison of glycemic index among all pasta samples.

Two Comparison Samples	Difference	Lower 95% CI	Upper 95% CI	*p*-Value
ICS-COMM	−3.7	−27.0	19.6	0.997
RU DIMESA-COMM **	**21.4**	**−2.0**	**44.7**	**0.092**
RU PONTE-COMM	18.4	−4.9	41.7	0.205
TI DIMESA-COMM	19.3	−4.0	42.6	0.164
TI PONTE-COMM *	**25.3**	**2.0**	**48.6**	**0.026**
RU DIMESA-ICS *	**25.1**	**2.2**	**48.0**	**0.024**
RU PONTE-ICS **	**22.1**	**−0.8**	**45.0**	**0.065**
TI DIMESA-ICS *	**23.0**	**0.1**	**45.9**	**0.049**
TI PONTE-ICS *	**29.0**	**6.1**	**51.9**	**0.005**
RU PONTE-RU DIMESA	−3.0	−25.9	19.9	0.999
TI DIMESA-RU DIMESA	−2.1	−25.0	20.8	1.000
TI PONTE-RU DIMESA	3.9	−19.0	26.8	0.996
TI DIMESA-RU PONTE	0.9	−22.0	23.8	1.000
TI PONTE-RU PONTE	6.9	−16.0	29.8	0.950
TI PONTE-TI DIMESA	6.0	−16.9	28.9	0.972

C.I.= Confidence Interval; * Marginal significant difference; ** Significant difference.

**Table 6:** For pasta sample ICS, COMM, RU-DIMESA RU-PONTE, TI-DIMESA, TI-PONTE tables (a–f) report the average glycemic response (mmol /L) after food ingestion, recorded at zero (t0), 15, 30, 45, 60, 90, and 120 min in enrolled volunteers; SD = Standard deviation; Var = Variance.

ICS (a)				COMM (b)			
T *Minutes*	Glyc *Mean*	SD	Var	T *Minutes*	Glyc *Mean*	SD	Var
0	5.26	0.33	0.11	0	5.20	0.55	0.31
15	5.79	0.58	0.34	15	5.80	0.59	0.35
30	6.35	0.51	0.26	30	6.31	0.78	0.62
45	5.97	0.57	0.33	45	5.86	0.69	0.47
60	5.8	0.71	0.51	60	5.90	0.93	0.87
90	5.81	0.49	0.24	90	5.58	0.49	0.24
120	5.65	0.51	0.26	120	5.42	0.41	0.17
**RU-DIMESA (c)**				**RU-PONTE (d)**			
**T** ***Minutes***	**Glyc** ***Mean***	**SD**	**Var**	**T** ***Minutes***	**Glyc** ***Mean***	**SD**	**Var**
0	4.90	0.36	0.13	0	5.30	0.35	0.12
15	5.57	0.58	0.34	15	5.90	0.53	0.28
30	6.69	0.79	0.63	30	7.34	0.49	0.24
45	6.64	0.83	0.69	45	6.92	1.09	1.19
60	6.18	0.97	0.94	60	6.32	0.94	0.88
90	5.72	0.65	0.43	90	5.81	0.56	0.31
120	5.56	0.57	0.32	120	5.61	0.60	0.36
**TI-DIMESA (e)**				**TI-PONTE (f)**			
**T** ***Minutes***	**Glyc** ***Mean***	**SD**	**Var**	**T** ***Minutes***	**Glyc** ***Mean***	**SD**	**Var**
0	5.15	0.5	0.25	0	4.82	0.43	0.18
15	5.78	0.67	0.45	15	5.68	0.45	0.21
30	6.74	0.87	0.76	30	6.61	0.64	0.41
45	6.46	0.89	0.79	45	6.33	0.96	0.92
60	6.25	0.72	0.52	60	5.9	0.89	0.79
90	5.93	0.56	0.31	90	5.74	0.81	0.66
120	5.94	0.46	0.21	120	5.53	0.66	0.44

**Table 7:** For reference food (50 g of glucose in 250 mL mineral water) tables (g,h) report the average values recorded for glycemia (mmol/L), SD (Standard deviation), and Var (Variance) in enrolled volunteers. Reference food was tested twice in two different sessions (Reference 1/Reference 2).

REFERENCE 1 (g)				REFERENCE 2 (h)			
T *Minutes*	Glyc *Mean*	SD	Var	T *Minutes*	Glyc *Mean*	SD	Var
0	5.16	0.43	0.18	0	5.19	0.38	0.15
15	6.82	0.98	0.97	15	6.79	1.03	1.07
30	8.38	1.31	1.71	30	8.21	1.25	1.57
45	8.66	1.53	2.35	45	8.02	1.51	2.29
60	8.24	1.45	2.11	60	7.67	1.08	1.16
90	6.03	1.13	1.29	90	5.45	1.05	1.11
120	5.06	1.24	1.53	120	4.65	0.93	0.87

**Table 8:** One-way ANOVA test (six pasta sessions and two reference food sessions).

Time	*p*-Value
0	0.095
**15**	**<0.001**
**30**	**<0.001**
**45**	**<0.001**
**60**	**<0.001**
90	0.743
**120**	**0.002**

## Data Availability

The datasets generated for this study are available on request to the corresponding author.
